# Developing Danish general practice

**DOI:** 10.3109/02813432.2013.815989

**Published:** 2013-09

**Authors:** Jørgen Nexøe

**Affiliations:** Associate Professor, General Practitioner, Research Unit of General Practice, University of Southern Denmark, Odense, Denmark. E-mail: jnexoe@health.sdu.dk

Conflict of interests

The Association of Health Authorities, Danish Regions, has refused to renew the current collective bargaining agreement after nine months of failed talks with the Organisation of General Practitioners. Subsequently, the Government announced a law that would force GPs to accept new working conditions if they wish to remain suppliers within the public healthcare system. In response the GPs have collectively decided to give up their provider number. A labour conflict with effect from September 2013 seems very likely. Thus the Danes may from September 2013 be denied free access to primary health care and Danish primary health care is at risk of collapsing.

General practice is in many ways the backbone or cornerstone of the Danish healthcare system, forming the front line of the healthcare system [1,2]. Nearly all Danish citizens (98%) are listed with a general practice [2]. The Danish healthcare system favours an interpersonal doctor–patient relationship and continuity of care, which are highly valued by the patients [3]. During the course of one year about 85% of all citizens are in contact with their general practitioner (GP) [4].

The current structure and position of Danish primary health care have developed over more than 100 years [5]. The ability to adapt to changing circumstances and challenges has ensured general practice an important position in the Danish healthcare system, providing cost-efficient, first-line services and careful gatekeeping [6].

Approximately 3600 GPs serve the Danish population and they make up about 20% of the physician workforce. The GPs are distributed across approximately 2200 practice units with one or two GPs in most practices. In principle, any doctor with a specialty in general practice can set up a surgery, but to receive reimbursement from the public authority a GP provider number must be granted for services to be free to patients. With few exceptions doctors own the practice units in which they work, meaning the doctors are self-employed. The GPs employ and pay nurses and other staff. Their main source of income is reimbursement. GPs are paid by a mixture of per capita payment and fees for services. Privately paid fees from patients or other services outside the National Health Service account for only a modest turnover [6].

GP offices are contracted to be open on all weekdays from 8:00 a.m. to 4:00 p.m., with the first hour reserved for telephone consultations. On one weekday, opening hours run to 6 p.m. or 7 p.m. The Regions now want longer hours to provide accessibility outside normal working hours without supplying any additional financing. The GPs are supposed to make efficiency gains as has been done in the hospitals, even though these efficiency gains are at least in part attained by transferring tasks to general practice. Furthermore, GPs will be forced to take over public health and preventive health tasks from the municipalities with no extra payment and without prior negotiations. It is proposed that patient care data from general practice, including diagnoses, prescriptions, laboratory tests, and information from hospitals, which until now have been used for quality improvement and research, will now also be used to monitor the individual GP.

The anticipated conflict may have a great impact not only on health care for patients. Development may be slowed down and research in primary health care may be halted for a period. In a situation with no agreement between the GPs and Danish Regions, data from the GPs’ electronic record system cannot be expected to be forwarded to the joint unit for quality development between the Organisation of General Practitioners and the Danish Regions. The risk of losing valuable data is substantial.

Negotiation rather than legislation is the solution, if we want to preserve effective primary health care in Denmark. Latest: on 29 June 2013 the Organisation of General Practitioners decided that the Danish general practitioners should not collectively give up their provider number. The chairman Henrik Dibbern has resigned. New acting chairman is Bruno Meldgaard Jensen. Negotiations between the Organisation of General Practitioners and the Danish Regions are resumed.
